# Does Basic Sanitation Prevent Diarrhea? Contextualizing Recent Intervention Trials through a Historical Lens

**DOI:** 10.3390/ijerph17010230

**Published:** 2019-12-28

**Authors:** Jesse D. Contreras, Joseph N.S. Eisenberg

**Affiliations:** Department of Epidemiology, University of Michigan, Ann Arbor, MI 48109-2029, USA; jdcon@umich.edu

**Keywords:** sanitation, diarrhea, diarrhoea, WASH, sewerage, intervention, meta-analysis

## Abstract

Three of four recent major sanitation intervention trials found no effect on diarrhea. These results conflicted with longstanding beliefs from decades of literature. To understand this discordance, we placed recent trials into the historical context that preceded them in two ways. First, we evaluated the history of published literature reviews on sanitation and diarrhea. Second, we conducted meta-analyses on studies from the most recent systematic review to uncover features that predict effectiveness. We found that 13 literature reviews dating to 1983 consistently estimated a significant protective effect of sanitation against diarrhea. However, these were marred by flawed studies and inappropriately averaged effects across widely heterogeneous interventions and contexts. Our meta-analyses highlight that the overall effect of sanitation on diarrhea was largely driven by sewerage and interventions that improved more than sanitation alone. There is no true overall effect of sanitation because variability between intervention types and implementation contexts is too complex to average. Ultimately, the null effects of recent latrine interventions are not surprising. Instead, the one trial that found a strong relative reduction in diarrhea is the historical outlier. The development of transformative sanitation interventions requires a better understanding of the social and environmental contexts that determine intervention effectiveness.

## 1. Introduction

Three recent and rigorously conducted intervention trials found that basic improvements to household sanitation had no effect on diarrhea among young children in Kenya (WASH-Benefits Kenya [1]), Zimbabwe (Sanitation, Hygiene, Infant Nutrition Efficacy trial (SHINE) [2]), and Mozambique (Maputo Sanitation trial (MapSan) [3]). A similar sanitation intervention did lead to a 39% decrease in the prevalence of childhood diarrhea in Bangladesh, from 5.7% to 3.5% per week (WASH-Benefits Bangladesh [4]). None of these interventions had an impact on child growth two years after the intervention.

These studies successfully tested specific hypotheses: providing or improving latrines at the household level prevents diarrhea and improves child growth among children in that household. However, as is true for all intervention trials, generalizability of these results to other interventions and settings is limited [5,6,7]. For example, these household-level trials did not test the effect of sanitation at high community coverage, which has been shown to be an important predictor of intervention effectiveness [8,9,10]. Due to this question of generalizability, it is important to assess how these results fit into the history of sanitation evidence, while acknowledging that these studies reflect some of the most thorough examinations of sanitation and diarrhea ever conducted. In 1991, a literature review found that sanitation interventions reduced diarrhea by 36% on average, a number widely cited over the following years [11]. The most recent systematic review of sanitation interventions found an overall diarrheal reduction of 25% [8].

Thus, it is useful to consider how the results of recent trials fit into the entire body of evidence. Before these trials, there was an evidence-based consensus that sanitation interventions prevented diarrhea. These recent data points do not negate years of experience; however, their relative high quality raises important questions. Why do the results from three of four of these trials disagree with previous estimates? Which effects should inform interventions and policy decisions?

One common feature of previous meta-analyses is that the average effect of sanitation has been estimated across widely heterogeneous groups of studies. Summarizing studies that measured different forms of sanitation, in different settings, and with different contextual factors obfuscates details on what is required to affect health. Some of these nuances have been noted, such as the stronger effect of sewerage interventions and interventions achieving high community coverage [8], but still questions remain on additional study features that characterize successful sanitation interventions.

To help answer these questions, we conducted a review of the historical evidence of sanitation effects on diarrhea, as well as a series of meta-regression analyses on intervention studies. Specifically, this review has two aims: (1) describe the historical evidence on the relationship between sanitation access and diarrhea by reexamining the history of literature reviews on the topic, and (2) characterize heterogeneity across results from all existing intervention studies to place more recent trials within a historical context and to identify features of successful interventions.

## 2. Methods

In the first aim, we evaluated the history of literature reviews on the relationship between sanitation and diarrhea from the earliest review identified (1983) to the latest (2018). We describe the group of studies included in each review, its conclusions and limitations, and conclude with a summary of how the prevailing estimate of the overall effect of sanitation on diarrhea has changed over the last three decades. For the second aim, we conducted sub-group meta-regression analyses on intervention studies identified in the most recent systematic review [8]. We categorized this list of studies on several factors, such as intervention type and coverage level, and included these as study-level covariates to demonstrate their effects on intervention success [12]. We describe features that may modify intervention effectiveness to a greater degree than previous reviews and identify the types of studies that drive historical expectations of an effect of sanitation on diarrhea.

### 2.1. History of Literature Reviews

To review past literature on sanitation and diarrheal disease, we conducted a systematic search to identify all literature reviews on the topic. We searched PubMed and Embase using the following search terms: (diarrhea OR diarrhoea) AND (sanitation OR latrine OR sewer *). Each search term was restricted to the title, abstract, or author keywords. The search results from each database were restricted to reviews. We assessed the titles and abstracts from each search to identify reviews on the relationship between sanitation and diarrhea. Articles were excluded if they were specific to a country, region, population (e.g., HIV patients), or infectious agent (e.g., cholera). Reviews were not included if they descriptively discussed the issue of diarrhea and/or sanitation without adding new information on their relationship. The references of each identified review were checked for additional reviews that were not identified by our initial search. Each identified review was assessed in detail to determine the types of studies reviewed and its conclusions. In addition, the cited references of each review were evaluated to better assess the strength of evidence included and to uncover caveats to its conclusions. We present a short description of our findings for each review in chronological order, along with a brief history of how the consensus estimate for the overall effect of sanitation on diarrhea changed over time (Table 1).

### 2.2. Sub-Group Meta-Regression Analyses

Heterogeneity among sanitation intervention trials was characterized through meta-regression analyses of studies identified in the latest systematic review (Table 2). Eligible studies were those that tested sanitation interventions, including randomized, quasi-randomized, and non-randomized controlled trials; case-control and cohort studies if they were related to a specific intervention; time-series studies; and cross-sectional household survey studies if they used an appropriate causal matching method (e.g., propensity score matching) [8]. The authors searched Pubmed, Embase, Scopus, and Cochrane Library for eligible studies between 1970 and 2016 and followed PRISMA (Preferred Reporting Items for Systematic Reviews and Meta-Analyses) guidelines. We created a list of studies reviewed by Wolf et al. (2018) from the article text and Supplementary Materials. The WASH-Benefits Bangladesh, WASH-Benefits Kenya, and SHINE trials were added to the final study list. The results of the MapSan trial were not included, as these were not publicly available during the completion of this review.

The text of each article was reviewed to understand the type of sanitation intervention, study design, and results of each study. After this initial review, we constructed a set of variables to extract from each study. The variables we selected were trial features that varied between studies and that could potentially modify the effect of sanitation interventions on diarrhea. The list of variables included (1) sanitation intervention type, (2) use of the community-led total sanitation (CLTS) model, (3) sanitation access initiation (i.e., whether the household made the decision to obtain sanitation or if the intervention was provided to households by the study team directly), and (4) community coverage.

We classified studies into categories of intervention type defined by four indicator variables: (i) latrine interventions, (ii) interventions that included more than sanitation (e.g., social capital or water quality interventions; but excluding hygiene promotion), (iii) sewerage interventions, and (iv) no intervention, which comprised causal analyses of national surveys or Demographic and Health Surveys (DHS).

Studies that employed CLTS methods were indicated with a binary variable. Two final indicator variables were created for household-initiated sanitation access and study-initiated interventions. Household-initiated sanitation access included interventions that promoted sanitation construction and offered free or subsidized facilities if the household was motivated to receive them without direct study contact, along with studies on existing sanitation access, such as from DHS and national survey data. Study-initiated intervention studies were those in which households were asked to participate with the knowledge that a sanitation facility would be constructed by the study team upon agreement. Sewerage studies were excluded from both groups.

Community coverage with the intervention was extracted from studies that measured total sanitation coverage among intervention communities after the intervention occurred, per the definition of sanitation used in the study. Coverage was extracted only if it was measured for the entire community. For example, if a study randomly selected a subset of households to receive the intervention and only reported that 100% of the sampled households received the intervention, no coverage value would be extracted. After extracting reported community sanitation coverage, this value was used to create additional indicator variables for various coverage thresholds. Wolf et al. showed a stronger effect of interventions that achieved ≥75% coverage compared to those that reached <75% of households, but this was the only threshold reported. To observe the range of potential threshold values, we created three indicator variables for coverage at or above 60%, 75%, and 90%, respectively. We chose 60% because it resulted in an even number of studies above and below the threshold while lower thresholds led to few studies below the threshold. We chose 90% in order to observe the effects of very high coverage. Because sewerage interventions inherently reach 100% coverage, we repeated the sub-group analyses by coverage after excluding sewerage studies to determine the impact of coverage on toilet or latrine-based interventions specifically.

Each of these indicator variables represent a potential modifier of the effect of sanitation interventions on diarrhea. To test the impact of these effect modifiers, meta-regression models were constructed to estimate a pooled effect of interventions within each category. For example, an average effect was calculated for the subset of studies that had a value of 1 for the sewerage indicator variable. These sub-group estimates were compared to each other and to the overall effect of all studies to assess which variables modify intervention effectiveness. But because average effects can conceal important differences between studies, we described the characteristics of individual studies in Table 2 and constructed a forest plot to show their individual effects. We further describe key differences within some sub-groups in the closing discussion.

Study estimates and 95% confidence intervals (CIs) were extracted from the Supplementary Materials of Wolf et al. (2018) to take advantage of the conversion to risk ratios (RRs) the authors already completed. Meta-regression models were fit using the *metafor* package in R [12]. For all models, study estimates were weighted by their inverse standard error, which was calculated as

1/[(RR_upper_ 9 − RR)/1.96],

where RR_upper_ is the upper RR of the 95% CI and 1.96 represents the critical *z*-score at the 95% confidence level.. Models were fit with random effects in order to match the methods used by Wolf et al.

## 3. Results

The systematic search for literature reviews on sanitation and diarrhea resulted in 199 possible reviews. After reviewing the titles and abstracts of these 199 results, 164 articles were deemed not relevant to the relationship between sanitation and diarrhea or were focused on a specific population. Of the remaining 35 articles, 10 literature reviews were identified on the relationship between sanitation and diarrhea [8,11,15,25,26,40,41,47,54,72]. Fifteen articles were excluded because they did not review primary literature; these articles or book chapters described the topic of sanitation and/or diarrhea broadly and cited other reviews if numeric estimates were present. Another eight articles were excluded because they did not specifically review sanitation and diarrhea together, e.g., if the outcome of interest was enteric dysfunction or the review focused on clinical care. One likely relevant study was excluded because it was published in Portuguese [91]. One eligible review found only one study [55]; we did not include this article in our analysis as it is not clear why the authors did not find a number of eligible studies that were found in earlier reviews [92]. After searching the references of the 10 identified literature reviews, three additional reviews were identified resulting in a total of 13 literature reviews on the relationship between sanitation and diarrhea [13,14,18].

### 3.1. History of Literature Reviews

#### 3.1.1. Blum and Feachem, 1983

The first literature review we describe was published by Blum and Feachem in 1983. This review referenced one earlier review conducted by a scientific working group of the WHO in 1979, but the text available online omits the relevant pages describing evidence on health outcomes [93]. Blum and Feachem identified studies that assessed the relationship between water supply and/or excreta disposal facilities on any health outcome. Health outcomes included diarrhea and/or dysentery, enteric infection, nutritional status, eye or skin infection, and mortality. But instead of summarizing the health effects of these studies, Blum and Feachem focused on the severe methodological limitations they found in the literature. The authors found that even though most studies claimed to show health improvements, methodological problems raised “serious doubts as to the validity of their conclusions” [13].

The authors focused on 44 published studies of water supply or sanitation and diarrhea or diarrhea-related infection. They found seven primary methodological problems: lack of adequate control (having no control group or a non-comparable control), the one to one comparison (comparing only one exposed village to another unexposed village), confounding variables, health indicator recall (they considered any recall period over 48 h as a methodological problem), health indicator definition, failure to analyze by age, and failure to record facility usage. Fourteen of the 44 studies measured diarrhea as an outcome and included sanitation in their exposure assessment, including three studies conducted in the United States in the 1950s and 1960s. The review does not separate studies that measured sanitation in isolation from those that studied water supply and sanitation together.

Additional study details are not described here, as the focus of the review by Blum and Feachem was on the severe limitations of these studies. Only one study out of the 44 was found to have none of the seven major methodology problems: a cross-sectional analysis of sanitation and helminth infections in Tennessee [94]. The remaining 43 studies had at least one severe limitation, and most had multiple methodology problems. The most common problem was the lack an “explicit effort” to control for important confounding variables [13]. Only seven studies were found to have adequate control for confounding variables, including three on sanitation and diarrhea [95,96,97]. Overall, the authors concluded that there was little confidence on the health effects of sanitation, despite the number of studies conducted on the topic. They emphasized the importance of understanding the health benefits of improved water and sanitation access by the end of the International Drinking Water Supply and Sanitation Decade (1981–1990).

#### 3.1.2. Esrey and Habicht, 1986

The first of two reviews led by Esrey aimed to evaluate the effect of water and sanitation interventions on diarrheal disease, infection, nutritional status, and childhood mortality from studies conducted after 1950. The authors note the importance of randomly allocating WASH (water, sanitation, and hygiene) interventions but did not limit the review to randomized trials or even intervention studies. The review included any study that compared two or more groups with different water and/or sanitation conditions. Eight studies were identified that examined water and sanitation together without estimating their individual effects. Six of the eight found that sanitation was associated with improved health, although three were described as having serious study flaws.

Twenty-three other studies measured the association between sanitation access and disease, infection, or mortality. Eighteen of these studies reported an association between sanitation and improved health. Three of the 18 studies that found health improvements were described as having significant methodological flaws. Of the remaining 15 studies, only three could be confirmed as including the relationship between diarrheal morbidity and sanitation [98,99,100]; most of the remaining studies measured infant mortality. Only one of the three studies on diarrheal morbidity and sanitation found an association when comparing families with a pit toilet to families with no toilet [98], although none of the three studies controlled for any potential confounders. Esrey and Habicht concluded that sanitation interventions could help improve child health, especially when interventions are tailored to the local community, but did not attempt to estimate an overall effect of sanitation.

#### 3.1.3. Esrey et al. 1991

In 1991, Esrey and colleagues published another review that estimated the effect of drinking water and sanitation interventions on diarrheal disease, nutritional status, mortality, and infection with *Ascaris lumbricoides*, *Dracunculus medinensis*, hookworm, *Schistosoma haematobium*, *S. mansoni*, and trachoma. For diarrheal disease, the authors only searched for studies published after the previous review. An estimate of the overall reduction of diarrheal disease morbidity associated with sanitation improvements was calculated as the median value for all studies considered, rather than the mean.

Thirty studies on sanitation were included in this review, but the total number of studies that measured diarrhea as the outcome is not stated. Eleven studies that measured diarrhea and had an extractable effect estimate were included in an overall estimate for sanitation and diarrhea. The median reduction in diarrheal morbidity from these 11 studies was 22%. Five of these 11 studies were described as “rigorous” studies, indicating that they did not have serious methodological flaws. A separate overall estimate was calculated for “rigorous” studies. The five studies had a median diarrhea reduction of 36%. However, the studies considered “rigorous” or “flawed” were not defined in this review. Knowing which studies were included in the overall estimate is necessary to understand its limitations. Since the authors chose to summarize the studies with the median effect, which provides less information than a pooled estimate with a confidence interval, it is especially important to see the range of effects and determine how well the median represents this range. As we will discuss, the importance of these limitations is underlined by the persistence of this estimate over the next two decades.

#### 3.1.4. Fewtrell et al. 2005

Acknowledging the earlier reviews by Esrey and colleagues, Fewtrell et al. sought to create the first systematic review of water, sanitation, and hygiene interventions and their relative effects on diarrheal disease. This review was the first to focus specifically on studies assessing interventions and the first to model an overall effect through meta-regression. The authors searched for studies published before 26 June 2003 and used Esrey et al.’s previous reviews to identify additional eligible studies.

Only four studies were deemed eligible from the authors’ search. Two of these presented data that could be used to conduct a meta-analysis [16,17]. The other two studies are not identified in the text or any supplemental material. One study by Azurin and Alvero was an evaluation of communal latrines combined with improved water supply and their effect on the risk of cholera for people of any age (RR = 0.32, 95% CI 0.24, 0.42). The authors did not measure diarrhea as an outcome and did not control for potential confounders. Fewtrell and colleagues graded the study as “poor quality”. The other study by Daniels et al. measured the impact of a government latrine construction program on diarrheal disease using a hospital-based case-control study design (OR = 0.76, 95% CI 0.58, 1.01). Neither of these studies is a strong examination of the effect of sanitation on diarrhea. Despite identifying only two eligible studies, one of “poor” quality that measured cholera as its outcome, the authors calculated a pooled estimate and reported a 32% overall reduction in diarrhea associated with sanitation interventions (RR = 0.68, 95% CI 0.53, 0.87).

#### 3.1.5. Waddington et al. 2009

Waddington, Fewtrell, and colleagues updated their previous systematic review [15] a few years after its release and searched for studies published after 26 June 2003. Eligible studies were RCTs or those employing quasi-experimental designs, including matched analysis of survey data. Risk ratios, rate ratios, odds ratios, and prevalence ratios were recorded and used to calculate an overall estimate without conversion to a single ratio type. The authors instead ignore the potential overestimation of odds ratios and report the estimate from each study as its “effect size (ES)”. An overall ES was calculated as a weighted mean of each study’s ES without conversion.

The authors identified six studies that estimated the impact of sanitation on diarrheal disease [19,20,21,23,24]. None of these studies appeared in the previous review. One of the six studies was a large national survey of “poor” quality that used a diarrheal recall period greater than two weeks [22]. One study was deemed poor because the comparability between treatment groups was not clear in the text [23]. Another study that measured the effect of a large national latrine project in Honduras was described as poor because it used a one-month recall period and had unclear comparability between treatment groups [24]. The three high quality studies included a propensity score matched analysis of DHS data in Nepal [19] and two non-randomized studies of urban sewerage [20,21]. Using all six identified studies, the authors estimated an overall reduction in diarrheal disease of 37% (ES = 0.63, 95% CI 0.43, 0.93). This estimate was similar to their previous estimate (32%) and nearly identical to the 1991 estimate from Esrey et al. (36%), although the limitations of each already have been described.

#### 3.1.6. Clasen et al. 2010

In 2010, Clasen et al. published a new systematic review on sanitation interventions and diarrhea [25]. Their review described the four reviews that preceded it and aimed to apply a more rigorous search strategy using the methodology defined by the Cochrane Collaboration for systematic reviews. Clasen et al. included randomized, quasi-randomized, and non-randomized controlled trials of sanitation interventions. The authors found 13 studies that met these criteria, including seven studies published in Chinese [101,102,103,104,105,106,107], five published in English [49,58,108,109,110], and one in French [48]. There was no overlap in the studies identified in Fewtrell et al. (2005) or Waddington et al. (2009) and this review. Clasen et al. thoroughly described the types of interventions studied, potential sources of bias, and other characteristics of each study. The types of interventions varied, including unimproved latrines, shared latrines, improved latrines, biogas reactors, septic tanks, and relocating toilets “away from water sources”. Some information could not be extracted from many studies, especially from the eight non-English studies, such as baseline sanitation access, the type of water supply, intervention coverage, and risk of bias.

All of the identified studies were non-randomized controlled trials. Eleven out of the thirteen studies reviewed found that the sanitation intervention reduced diarrhea, but confidence intervals were only calculated for two studies [58,104]. Clasen et al. did not calculate confidence intervals for the other eleven studies due to insufficient number of intervention clusters (i.e., villages, communities, or schools). RRs for the effect of the intervention on diarrhea ranged from 0.20 [102] to 1.03 [108]. The authors concluded that sanitation interventions are effective at preventing diarrhea, but they did not estimate an overall effect of sanitation due to limited evidence. Clasen et al. described substantial heterogeneity in the existing literature that limited study comparability. They also note that only five of the 13 studies studied interventions of sanitation alone, without drinking water or other improvements, and that these five studies included limited geography. Four took place in China, and one was conducted in the United States [109].

Of the five English studies, two were later included in the most recent systematic review by Wolf et al. (2018) [49,58], along with the French language study [48]. Of the three English studies excluded from the most recent review, one was likely excluded due to measuring diarrhea from healthcare records [110] and one may have been excluded for its use of a borehole latrine intervention [109]. The other may have been excluded due to the authors reporting issues in implementation leading to low compliance [108]. None of the Chinese studies were included in the most recent review.

Clasen et al. (2010) conducted the most methodologically rigorous review between, at least, 1983 and 2014. However, they did not extract confidence intervals from 11 studies that included 1–3 clusters (e.g., villages) per intervention arm, instead only extracting point estimates. This decision limited the review’s analysis of intervention effects and impedes a clear understanding of each study’s results.

#### 3.1.7. Norman et al. 2010

Another review published in 2010 focused on the effects of sewerage on diarrhea [26]. This review was not limited to interventions, including both observational and intervention studies. Norman et al. found 25 studies that met these criteria, including six cohort studies, four case-control studies, one non-randomized intervention study, and fourteen cross-sectional studies. Fourteen of the 25 studies were conducted in Brazil, three took place in Mexico, and the remaining came from Nicaragua, Honduras, Peru, the United States, Iran, Syria, Saudi Arabia, and Australia. Diarrhea was the primary outcome of 17 studies, with the remaining eight studies measuring enteric infection [20,21,22,24,27,28,29,30,31,32,33,34,35,36,37,38,39]. Norman et al. estimated a pooled effect of sanitation on all outcomes from 25 studies (RR = 0.70, 95% CI 0.61, 0.79) and on diarrhea from 17 studies (RR = 0.70, 95% CI 0.58, 0.85). The authors noted that confounding is a potential issue with the inclusion of mostly observational studies. However, they showed that the effect of sewerage was even stronger for studies that included multivariate regression (RR = 0.64, 95% CI 0.53, 0.77) compared to studies that did not (RR = 0.78, 95% CI 0.63, 0.97). They also conducted a sensitivity analysis to show that even if there were a very strong unidentified confounder (RR with disease = 0.65; RR with exposure = 2.00), the RR for sewerage on diarrhea would still be 0.78. The types of studies included in this review were varied, and the study designs are not ideal for measuring a causal relationship. But the relationship between sewerage and diarrhea was consistent across all subgroup and sensitivity analyses, providing additional strength to the conclusions of Norman et al. that sewerage is associated with reduced diarrhea.

#### 3.1.8. Cairncross et al. 2010

Cairncross et al. sought to provide more information to the “consensus view on the impacts of health of improved water quality, water quantity and sanitation” established by Esrey and colleagues in their earlier reviews [11,14]. The authors again searched for intervention studies that measured the effect of sanitation on diarrheal disease. The search included articles published any time before April 2007. Cairncross et al. initially identified seven quasi-randomized intervention studies, but all of these included water quality interventions that precluded estimating the effect of sanitation alone. An additional search was conducted to identify more studies, and that search resulted in four new studies that were conducted in China and published in Chinese [101,103,104,105]. These were included in the seven Chinese studies reviewed by Clasen et al. (2010). The four studies estimated diarrheal reductions of 63%, 51%, 20%, and 8%, but confidence intervals were not shown. Finally, the scope of the review was widened to include before and after studies of sanitation, and one additional study was identified [34]. In this last study, diarrheal disease was measured before and after expansion of sewerage in Salvador, Brazil. The study found positive effects, as diarrheal disease was reduced citywide by 21% (95% CI 19%–26%) and by 43% (95% CI 39%–46%) in high-risk areas.

The authors decided not to calculate an overall estimate of sanitation interventions on diarrheal disease due to high variability in the types of interventions tested in the five studies. However, the authors still noted the “striking consistency between the reductions found in various reviews of 36% [11], 32% [15], 20%–51% (the four Chinese studies) and 22%–43% [34]”.

But there are several issues with this statement. The comparison excluded two of the four Chinese studies, which had reductions of 8% and 63%. This was likely to show the median effect of the four studies, but still obscures the wide range of estimated values and assumes the true value lies somewhere in the middle. In addition, the authors failed to note that one estimate is a single before and after analysis of urban sewerage [34] and that another estimate comes from only two studies [15]. The authors concluded that “there is not enough evidence to justify a departure from the prevailing consensus, published nearly two decades ago and widely cited with approval since then, that sanitation reduces diarrhoea risk by about 36%”.

Thus, our understanding of the impact of sanitation on diarrhea did not improve much between 1983 and 2010. A median estimate from 1991, based on five studies that we could not identify, remained the consensus. Other reviews were conducted, but these also were based on few studies and were indiscriminate on study quality and sanitation definition.

#### 3.1.9. Heijnen et al. 2014

A review published in 2014 by Heijnen et al. examined how shared sanitation compares to individual household latrines in preventing a number of health outcomes, including diarrhea, helminth infections, enteric fevers, other fecal-oral diseases, trachoma, and adverse maternal or birth outcomes [41]. Eligible studies compared these outcomes between individuals using shared sanitation and those using household latrines, with no limits placed on study design. Nine studies were found that compared this effect on diarrheal disease, and six had effect estimates available for inclusion in a meta-analysis. All six studies employed a case-control design and enrolled cases from health clinics, emergency departments, or hospital records. One of these studies was a multi-country analysis and contributed seven effect estimates to the meta-analysis, resulting in 12 total estimates ref. [45]. Compared to individual household latrines, shared sanitation was associated with a 44% average increase in the odds of diarrhea (OR = 1.44, 95% CI 1.18, 1.76). The types of shared sanitation included both communal latrines and household latrines that were shared between two or more families. Heijnen et al. completed a thorough review of the existing literature, but their analysis highlights the limited evidence on shared sanitation. The authors note that the underlying evidence allows for only weak causal inference and call for more research to determine if circumstances exist in which shared sanitation can be an effective tool for improving health.

#### 3.1.10. Wolf et al. 2014

The number of articles on sanitation interventions grew rapidly after 2010. In 2013, the WHO convened a meeting of experts to agree on protocols for new systematic reviews on WASH interventions and health outcomes. As a result of that meeting, Wolf et al. estimated the impact of drinking water and sanitation interventions on diarrheal disease [47]. This review included RCTs, quasi-randomized and non-randomized control trials with baseline data, case-control and cohort studies when they were related to an intervention, time-series studies, and observational studies using specific matching methods (e.g., propensity score matching). Studies were excluded if they were targeted to institutions, such as schools and workplaces, if they were conducted in non-representative populations, such as HIV patients, or if they had very low compliance (<20%). The search was limited to interventions occurring in low- and middle-income countries and studies published between 1970 and May 2013.

Eleven eligible sanitation studies were identified. Overall, sanitation interventions reduced diarrhea risk by 28% (RR = 0.72, 95% CI 0.59, 0.88). The effects of sewerage interventions were found to be substantially higher at 69% and 63%, but there were only two sewerage studies to compare [21,22]. The authors noted that this sample size is extremely limited and that the estimates should be treated with caution. Studies that measured a non-sewerage sanitation intervention led to a more modest, but significant, reduction in diarrheal disease of 16% (RR = 0.84, 95% CI 0.77, 0.91). This marks the first review that distinguished between the large effects of sewerage from the effects of other sanitation interventions, although all studies were included in the overall estimate of a 28% reduction.

#### 3.1.11. Jung et al. 2017

The role of neighborhood level sanitation in preventing diarrhea was reviewed by Jung et al. in 2017 [54]. Importantly, this review was not on neighborhood level coverage with household sanitation. Instead, the authors defined neighborhood sanitation as “the removal of exposed fecal matter or wastewater from the neighborhood”. This definition includes studies on sewerage or drainage access, the elimination of open defecation, or observations of neighborhood fecal contamination (e.g., presence of wastewater or fecal matter). In contrast, household sanitation was defined as “the presence of any type of household sanitation facility within the subject’s residence, or the disposal method of child feces”. The authors did not exclude any study designs. Studies were excluded if they reported an aggregate measure of neighborhood or household sanitation but did not control for sanitation at the other level, e.g., studies on sewerage that did not separate the effect of improved household sanitation. Thirteen studies were excluded for this reason, but the authors did not identify the excluded studies.

Twenty-two eligible studies were identified, including five studies on neighborhood sanitation, 16 studies on household sanitation, and one study that included estimates of both. Only five of these studies have been included in other reviews that we describe in this article [17,21,49,55,58]. The remaining studies all employed a case-control or cross-sectional design. Six studies on neighborhood sanitation found that the exposure was associated with 44% lower odds of diarrhea on average (OR = 0.56, 95% CI 0.40, 0.79), including significant effects in five of the six studies. The exposures of interest included “no sewage spillage around house”, “no observable feces in the neighborhood yard”, “no open sewage ditch nearby”, “no rubbish and fecal material lying around, blocked open drains around home and nearby streets”, “no wastewater in street”, and “communities with simplified sewerage and surface drainage vs. surface drainage only”. Household level sanitation was associated with 36% lower odds of diarrhea on average (OR = 0.64, 95% CI 0.55, 0.75). This association was nearly identical when divided between studies on the presence of sanitation and studies on children’s usage of sanitation facilities.

Jung et al. concluded that both neighborhood and household level sanitation is associated with decreased diarrhea, and that the magnitudes of each association are comparable. The article is limited in including almost exclusively observational research, but a review of observational evidence is a useful addition to other reviews that focus on intervention studies alone. The review is unable to assess whether the underlying associations were due to confounding, which is particularly important as the authors reported that eight studies did not adjust for likely confounders. The neighborhood level analysis is further limited by the definition of neighborhood sanitation. The exposures used in these studies, mostly relying on visual inspection for fecal matter, were not strong indicators of neighborhood sanitation. In addition, the strongest effect in this group was associated with a sewerage intervention and is not comparable to the other neighborhood level studies [21].

#### 3.1.12. Freeman et al. 2017

Freeman and colleagues conducted another WHO commissioned review of sanitation interventions and their effect on diarrheal disease, as well as helminth infections, trachoma, schistosomiasis, and nutritional status. Freeman and colleagues also aimed to update other reviews on soil-transmitted helminth (STH) infection, trachoma, schistosomiasis, and nutritional status. It is not clearly stated which eligibility requirements were employed for the review of diarrheal disease. Freeman et al. included most of the same studies as Wolf et al.; however, this review also included some non-intervention studies and school-based interventions that would have been ineligible in Wolf et al. 2014.

A total of 33 eligible studies were identified, and 27 were included in a meta-analysis. Of these 27 studies, 11 were included in Wolf et al. 2014. Three were studies on sewerage. Effect estimates were converted to ORs for meta-analysis. Using all 27 studies, Freeman et al. estimated that sanitation improvements reduce diarrhea by an average of 12% (OR = 0.88, 95% CI 0.83, 0.92). This estimate demonstrates a considerably smaller effect compared to previous reviews. However, this overall estimate included non-interventions that were previously ineligible, such as hospital-based case-control studies [84,85,86]. Sixteen studies were found that measured the effect of a sanitation intervention [21,22,24,49,50,55,73,74,75,76,77,78,79,80,81,82]. In a sub-analysis, these intervention studies were found to reduce diarrheal disease by 23% (OR = 0.77, 95% CI 0.66, 0.91). This estimate includes three studies (with five total effect estimates) on school-based sanitation interventions [75,76,82].

Freeman et al. also described the impact of sanitation coverage on intervention effectiveness. Of the 16 intervention studies, nine were described as reporting on latrine coverage or latrine use. Three of those nine studies found that the intervention reduced diarrhea. However, two of these studies were actually sewerage interventions [21,34]. The other study found that the intervention did not lead to increased latrine coverage, suggesting that latrine access did not reduce diarrhea. Instead, the authors attributed the reduction in diarrhea to drinking water and handwashing behavior [73] Thus, only sewerage studies appeared to have effects at high coverage.

Freeman et al. estimated an overall diarrheal reduction of 12%, but this estimate included a number of studies with non-generalizable designs, such as hospital-based case-control studies. Their estimate for the 16 intervention studies, a 23% reduction, is more in line with the results of previous reviews. However, this estimate still includes school-based interventions, which likely follow unique transmission dynamics, and three sewerage studies that possibly drive the observed overall effect of sanitation interventions.

#### 3.1.13. Wolf et al. 2018

While Freeman et al. focused specifically on sanitation and included several infection-related outcomes, Wolf et al. again reviewed the evidence on the impact of drinking water and sanitation interventions on diarrheal disease, with a new review on the effect of handwashing interventions [8]. This review was a direct update to Wolf et al. 2014 and used the same protocol. Unlike in Freeman et al. 2017, only intervention-based studies were eligible for inclusion. Observational study designs were allowed if they were conducted around an intervention. The search for new studies included articles published between January 2012 and February 2016, bringing the total range of studies to between 1970 and 2016.

In this update, eight new eligible sanitation studies were identified and added to the 11 studies from Wolf et al. 2014 [19,21,22,24,48,49,50,51,52,53,55,58,77,78,79,87,88,89,90]. Four estimates were extracted from Capuno et al. 2011, resulting in 22 total effect estimates from 19 studies. Using all 22 estimates, the overall effect of sanitation was estimated as a 25% reduction in diarrhea risk (RR = 0.75, 95% CI 0.63, 0.88). The authors again estimated the effects of sewerage interventions and non-sewerage studies separately. Two studies compared a sewerage intervention to a baseline of unimproved sanitation (RR = 0.60, 95% CI 0.39, 0.92) and two studies compared sewerage interventions to a baseline of improved sanitation (RR = 0.71, 95% CI 0.47, 1.07). Using 15 studies, the overall effect of non-sewerage interventions was a 16% reduction in diarrheal disease (RR = 0.84, 95% CI 0.73, 0.98), which is the same point estimate as found in Wolf et al. 2014.

The authors examined the impact of several study factors on the effect of sanitation interventions by including covariates in meta-regression models. The effect of sanitation interventions was not different when baseline access was unimproved sanitation versus open defecation. Access to an improved vs. unimproved water source, provision of a latrine vs. promotion only, survey data analyses, and follow-up time were found to be not associated with the effect of sanitation interventions on diarrheal disease. Combined interventions were found to be more successful than single interventions (RR = 0.59, 95% CI 0.43, 0.81). The authors then examined the effects of community coverage on intervention effectiveness. Twelve studies had available data on coverage after the intervention. Interventions that led to sanitation coverage of <75% reduced diarrhea by an average of 24% (RR = 0.76, 95% CI = 0.51, 1.13), and those that led to coverage >75% reduced diarrhea by 45% (RR = 0.55, 95% CI 0.34, 0.91).

Wolf and colleagues have provided the most thorough understanding of the evidence on sanitation and diarrheal disease to date. Unlike earlier reviews, the authors spend considerable attention to the unique study characteristics that lead to successful sanitation interventions. The review highlights that sewerage studies and studies that achieve high levels of sanitation coverage are much more successful at preventing diarrheal disease. However, the authors do not acknowledge that only five studies achieved coverage greater than 75%, and three of these were sewerage studies. The other two studies included a water, sanitation, and hygiene intervention [49] and a national sanitation intervention deemed poor quality in Waddington et al. 2009 [24]. Both found that the intervention resulted in lower diarrhea, but evidence on the effect of non-sewerage sanitation interventions at high coverage is limited. In addition, studies testing an intervention that included more than only sanitation reduced diarrheal disease 41% more (95% CI 19%, 57%) than studies with sanitation alone. This suggests that non-sanitation components of combined interventions could be driving the overall estimate of the effectiveness of sanitation, but these effects were not separated by Wolf et al. For their primary result, the authors chose to report the overall effect of sanitation interventions using all eligible studies: a 25% reduction.

#### 3.1.14. Updates to the Overall Effect of Sanitation over Time

For many of these historical reviews, estimating an overall effect of sanitation on diarrhea was the primary aim. It is useful to have a simple number to use in advocating for sanitation interventions, but the resulting effect estimates have obscured the fact that different sanitation interventions lead to different results. Realistic expectations for the success of WASH interventions should be based on more nuanced estimates for that type of intervention and, when possible, for specific contextual and study factors that apply to the intervention in question.

Despite the limitations of using one overall estimate to describe the effect of sanitation interventions, our understanding of these effects has clearly grown over time. The estimate from Esrey et al. in 1991 was “widely cited” and carried through to 2014 despite its limited conclusiveness as a median effect from only five unidentified studies. Two additional reviews were conducted but found very little new information [15,40]. One other review found six new studies, but graded half of these as poor quality [18]. The three high-quality studies included a national survey and two non-randomized sewerage studies. The overall effect estimate calculated in this review was very similar to the prevailing consensus, with an average reduction in diarrhea of 37%. Three reviews on specific components of sanitation found protective effects of sewerage, household latrines compared to shared sanitation, and neighborhood sanitation [26,41,54].

In 2014, Wolf and colleagues conducted a thorough review after a sizable growth in the number of available studies. Eleven intervention studies were reviewed and found an average reduction in diarrheal disease of 28% (RR = 0.72, 95% CI 0.59, 0.88). For the first time, the authors noted that two sewerage studies led to drastically larger reductions in diarrheal disease (69% and 63%) compared to the 16% reduction seen in non-sewerage studies (RR = 0.84, 95% CI 0.77 0.91). With a broader set of eligibility criteria, Freeman et al. updated the overall estimate of sanitation studies. They found a 12% average reduction in diarrheal disease (OR = 0.88, 95% CI 0.83, 0.92). When limited to only intervention studies, the authors found a more comparable reduction of 23% (OR = 0.77, 95% CI 0.66, 0.91).

Currently, the best estimate for the overall effect of sanitation comes from the latest review: Wolf et al. 2018. In this review, the authors found a similar reduction of diarrheal disease from sanitation interventions of 25% (RR = 0.75, 95% CI 0.63, 0.88). However, the authors again noted that the effect among non-sewerage studies was a more modest 16% (RR = 0.84, 95% CI 0.73, 0.98). Sewerage provision is still largely considered infeasible or unaffordable to achieve universal access to sanitation [26,111,112]. For more common interventions, mostly latrines, a 16% reduction can be considered the best estimate for the effect of sanitation on diarrhea. However, as the second aim of our review shows, the best average effect still covers a wide range of sanitation interventions and requires a deeper examination to reveal the nuanced effects of sanitation on diarrhea.

### 3.2. Sub-Group Meta-Regression Analyses

#### 3.2.1. Recreating the Overall Estimate from Wolf et al. 2018

Our analysis of the *History of Literature Reviews* demonstrates that sanitation interventions are too varied to describe with a single average estimate. We estimated an average effect across heterogeneous studies, but only to confirm that our meta-regression models were similar to those fit by Wolf et al. We aimed to recreate their overall estimate of a 25% average reduction (RR = 0.75, 95% CI 0.63, 0.88). While excluding the WASH-Benefits and SHINE trial results, we estimated an overall effect that is slightly attenuated (RR = 0.77, 95% CI 0.64, 0.90). We refit this model with fixed effects and various random effects estimators to test if the observed difference was due to model specifications, but the result was consistent across estimators. The disagreement could be due to the use of different weighting calculations, statistical programs, or subtle changes between the RRs reported in the text of Wolf et al. (2018) and those used in final analyses. Despite the small discrepancy, we assumed that our model results are similar to those that would be obtained directly by Wolf et al. using the same criteria. Due to the high degree of heterogeneity within these studies, the effect we estimated is not meaningful and only serves to test our methods against the original source.

#### 3.2.2. Intervention Type

Average estimates and confidence intervals for the effect of sanitation were calculated for the four intervention types described above: (i) latrine interventions, (ii) interventions that included more than sanitation alone (e.g., social capital or water quality interventions; but excluding hygiene promotion), (iii) sewerage interventions, and (iv) no intervention (causal analyses of national DHS surveys (Table 3).

**Figure 1:**
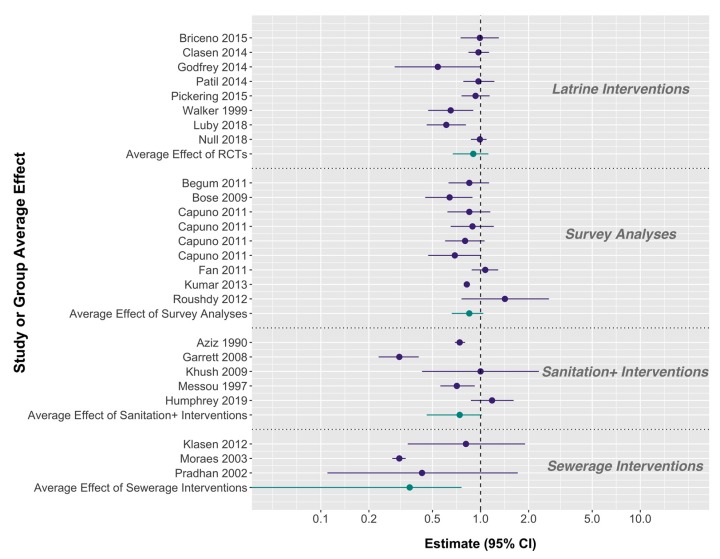
Forest plot of sanitation studies included in meta-analysis by intervention type. Effect estimate and 95% confidence intervals are plotted for each study (purple) and for the pooled estimate of four intervention types (green). The four intervention types are latrine interventions (Latrine Interventions), no intervention: causal analyses of national survey data (Survey Analyses), interventions that improved more than sanitation alone (Sanitation + Interventions), and interventions on sewerage access (Sewerage Interventions).

Including WASH-Benefits Kenya and Bangladesh, eight latrine interventions had no statistically significant average effect on diarrhea risk (RR = 0.90, 95% CI 0.67, 1.12; [1,4,24,55,78,79,87,90]; Figure 1). The pooled effect of the six non-WASH-Benefits latrine interventions was about the same (RR = 0.90, 95% CI 0.61, 1.18). There were five studies that intervened on more than sanitation alone, including the SHINE trial. These studies reduced diarrhea by an average of 26% (RR = 0.73, 95% CI 0.46, 1.02). This result was almost identical when excluding the results from the SHINE trial. Nine causal estimates from national survey or DHS analyses resulted in an average diarrheal reduction of 15% (RR = 0.85, 95% CI 0.66, 1.04). Lastly, three interventions on sewerage access led to a 64% average reduction in diarrhea (RR = 0.36, 95% 0.00, 0.76). But one study with a small confidence interval around a large effect magnitude (RR = 0.31, 95% CI 0.28, 0.34) appears to drive this estimate [21]. The other two sewerage interventions found no effect on diarrhea, but their interpretations are limited by sample size (23 children in the intervention group of Pradhan et al. 2002 [22]) and study design (Klasen et al. estimated the effect of sewerage by comparing a water plus sewerage intervention to a water intervention, in two geographic regions that had opposite results [77]).

The studies that found the largest effect of sanitation on diarrhea were on sewerage (64% reduction), followed by those on interventions including more than sanitation alone (26%), and national survey or DHS data (15%) (Figure 1). Latrine interventions, whether considering the most recent trial results or not, did not have a significant effect on diarrhea on average. The studies included in each of these groups are similar on intervention type, but they still are characterized by a high degree of heterogeneity. Our pooled estimates help demonstrate broad differences between interventions and the severe limitations of estimating a single effect of sanitation, but these estimates still average effects across widely different contexts and require a more nuanced understanding of the studies described.

#### 3.2.3. Community-Led Total Sanitation

Four studies employed a CLTS model, each employing an RCT design [78,79,87,90]. These studies did not impact the risk of diarrhea in children on average (RR = 0.91, 95% CI 0.55, 1.28; Table 3).

#### 3.2.4. Initiation of Sanitation Access

Studies on sanitation access that was household-initiated had a stronger effect on diarrhea compared to study-initiated interventions (Table 3). Fifteen estimates from 12 studies on household--initiated sanitation led to a 16% average reduction in diarrhea (RR = 0.84, 95% CI 0.68, 1.00), while four study-initiated interventions did not have an effect on diarrhea on average (RR = 0.95, 95% CI 0.67, 1.24 [1,2,4,55]).

#### 3.2.5. Community Coverage

**Figure 2:**
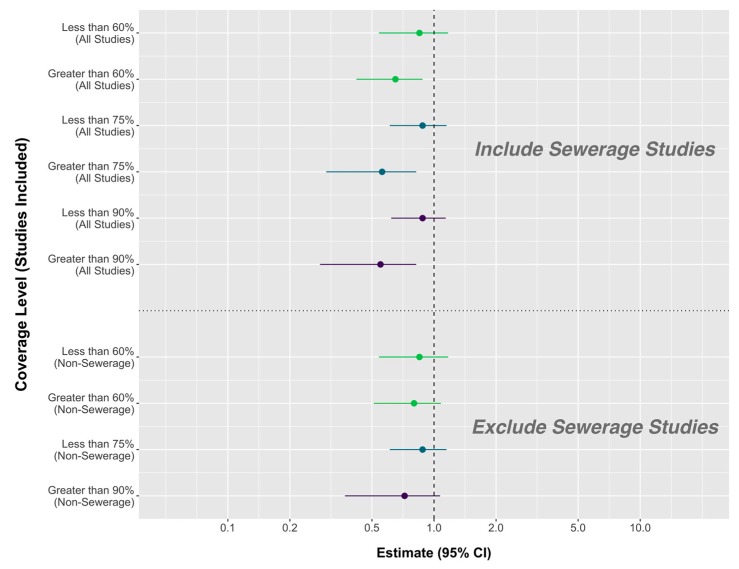
Forest plot of sanitation studies by community coverage with the intervention for (top) all studies and (bottom) non-sewerage studies. Effect estimate and 95% confidence intervals are plotted for three coverage thresholds: 60% (green), 75% (blue), and 90% (purple). No non-sewerage studies reached coverage between 75% and 90%.

Thirteen studies in this analysis had available sanitation coverage data. The WASH-Benefits and SHINE trials intervened in a subset of houses within a community and did not measure total coverage. Studies with higher community coverage had a larger effect on diarrhea using cutoffs of 60%, 75%, and 90% (Table 4; Figure 2). Studies that did not reach 60% coverage found no average effect (5 studies; RR = 0.85, 95% CI 0.54, 1.17). Studies that reached coverage over 60% reduced diarrhea by an average of 35% (8 studies; RR = 0.65, 95% CI 0.42, 0.88). Studies with a final community coverage under 75% had no significant effect overall (8 studies; RR = 0.88, 95% CI 0.61, 1.15), while studies with coverage over 75% reduced diarrhea by 44% on average (5 studies; RR = 0.56, 95% CI 0.30, 0.82). Lastly, studies that did not achieve 90% coverage again did not significantly impact diarrhea on average (9 studies; RR = 0.88, 95% CI 0.62, 1.14), but the strongest effect was found among studies that achieved coverage over 90%, with a 45% reduction in diarrhea risk (4 studies; RR = 0.55, 95% CI 0.28, 0.82). Only one study reached coverage above 75% but below 90% (85% coverage [77]), resulting in nearly identical results using the two cutoffs.

After excluding three sewerage interventions, only two remaining studies resulted in coverage over 75% [24,49]. Both studies also reached coverage over 90%, so models for the two cutoff values are the same. Eight studies that did not achieve 75% coverage again had no effect on diarrhea, while the two studies that achieved coverage at or above 90% resulted in a non-significant 28% average reduction in diarrhea (RR = 0.72, 95% CI 0.37, 1.07). The effect of coverage among non-sewerage interventions nearly disappeared at the 60% threshold. Five studies that did not reach 60% coverage led to a non-significant 15% average reduction (RR = 0.85, 95% CI 0.54, 1.17). The remaining five studies that did reach coverage over 60% resulted in a non-significant effect that was almost of the same magnitude (RR = 0.80, 95% CI 0.51, 1.08).

## 4. Discussion

Recently conducted sanitation intervention trials had no impact on child growth and most had no effect on diarrhea. The lack of an effect on diarrhea was particularly surprising against a backdrop of historical evidence that seemingly suggested sanitation is highly effective in its prevention. The WASH-Benefits trials aimed to assess whether combined interventions were “more effective than single interventions”, highlighting the prevailing expectation that water, sanitation, and hygiene alone would have an effect on diarrhea [113]. In the first part of this review, we showed that the null effects of sanitation on diarrhea found in Kenya, Zimbabwe, and Mozambique should not be as surprising as they first seemed (Figure 1). Instead, the strong effect of sanitation found in WASH-Benefits Bangladesh is the more surprising result. We found that prior estimates that sanitation reduces diarrhea by 23%–37% were based on averages that inappropriately included poorly conducted studies and combined widely different types of interventions, including latrines, sewerage, and those that included more than sanitation alone. These overall estimates have obfuscated the true effects of different sanitation interventions by masking the high degree of heterogeneity among studies. Some of the review authors attempted to describe these nuances, but the study features considered were limited and the authors still chose to report an overall effect of all study types as the primary result.

In the second part of this review, we more thoroughly disentangled this nuance in the current body of evidence and showed the limitations of summarizing the literature with a pooled estimate. We found that sewerage interventions drove the protective effect of sanitation estimated in the most recent systematic review, as did interventions that included more than sanitation improvements alone. Latrine interventions did not affect diarrhea on average. But a high degree of heterogeneity remains within each of these groups. Although most latrine interventions did not show an impact, three latrine-based interventions did reduce diarrhea. Even between the two recently conducted trials, discordant results were found. Sanitation had no effect on diarrhea in WASH-Benefits Kenya, while there was a 39% relative reduction found in WASH-Benefits Bangladesh.

Along with these large differences by intervention type, we found that two additional study features are important in predicting the effectiveness of a sanitation intervention: intervention coverage and household motivation to achieve sanitation access. Previous estimates have shown that high coverage with a sanitation intervention leads to larger reductions in diarrhea, but we found that this difference is substantially diminished after excluding sewerage interventions (Figure 2). For latrine interventions, reaching very high coverage (over 90%) may improve effectiveness, but this is only supported by one combined WASH intervention and one latrine estimate that is likely confounded [24,49]. Nonetheless, some prior observational studies do support a herd protection effect. There is stronger evidence within this review to support the increased effectiveness of sanitation when the household, rather than a study team, initiates access. Below, we discuss in detail the influence of: (1) sewerage decoupled from other types of sanitation interventions (2) latrine interventions, highlighting further heterogeneity and the limitations of average effect estimates, (3) intervention coverage and the potential for herd protection, and (4) the source of sanitation initiation, which might partially explain why many sanitation interventions fail to prevent diarrhea.

### 4.1. Sewerage Interventions

We found that the overall effect of sanitation was strongly influenced by sewerage interventions, which led to a 64% average reduction in diarrhea. However, these results are mostly based on one study in Brazil [21]. The other two sewerage studies do not provide clear information on how the intervention affected diarrhea. In Nicaragua, a complex social investment project did not find an effect [22]. However, not all households in the intervention area were connected to the sewer system, and only 23 children under six were measured in the intervention group. Two of those children were reported to have diarrhea. Another intervention expanded sewerage and piped water access in mountain and coastal regions of Yemen [77]. The control group for the sanitation intervention comprised households that received only the piped water intervention, limiting the reliability of the sewerage estimate. The effect of sanitation on diarrhea was negative in the coastal region and positive in the mountain region, but only the coastal effect was included in the latest systematic review. Thus, the effect of sewerage found in our meta-analysis was largely based on one study in Brazil, which greatly limits the generalizability of its conclusion. Additional support for an effect of sewerage on diarrhea was found in a sewerage-specific literature review, which found a 30% overall reduction in diarrhea associated with sewer access [26]. Sustainability and affordability are important limitations in expanding sewerage to achieve universal sanitation access. But its strong association with health, although from limited evidence, supports considering the example of sewerage when designing and implementing new sanitation interventions.

If connections are accessible, sewerage can reach universal coverage in the population and achieve the potential health benefits of herd protection. Functional sewerage infrastructure completely separates users from fecal waste without risk of exposure during pit emptying or from flies around pit latrines. These benefits underscore the utility of sewerage in reaching the Joint Monitoring Programme’s (JMP) definition of safely managed sanitation: the use of improved sanitation facilities that are not shared with other households and where excreta are safely disposed of in situ or transported and treated off-site [114]. However, sewerage may not be the best sanitation option in all settings, such as very rural communities or water-stressed regions. For these communities, new strategies are needed to safely manage sanitation without the same resource requirements.

### 4.2. Latrine Interventions

Eight latrine interventions (without additional intervention components) had no average effect on diarrhea. Three latrine interventions, including WASH-Benefits Bangladesh, did demonstrate an effect on diarrhea. One was a large-scale national sanitation campaign conducted in Honduras in the 1990s that measured diarrhea occurrence in all age groups using a one-month recall [24]. The intervention involved provision of World Bank funds to local municipalities, which were asked to choose a social investment project to have implemented. The options included items such as a new school, drinking water projects, or latrine construction, and were provided by local contractors. It is not clear if municipalities could only choose one project or if they could choose multiple projects within their budget. Waddington et al. rated this study as poor quality due to its use of a one-month recall period for diarrhea and because the “comparability of treatment and control groups [was] not sufficiently clear”. Control households in this analysis were “pipeline controls” that had not received the intervention, but would soon receive the intervention. The manuscript text does not explain if controls for the latrine analysis were those who had elected to receive the latrine project, or those that had not yet selected their project. The authors showed that the control group was more rural, less educated, had poorer access to baseline sanitation, and had less income compared to intervention communities. Walker et al. conducted multivariate regression to account for some of these differences, but that estimate was not used in the latest systematic review. Compared to households with a “washable toilet”, households with no access to sanitation facilities had higher odds of diarrhea (Odds Ratio (OR) = 2.68, *p* = 0.05). The definition of a washable toilet was not provided, but we believe it indicates a porcelain toilet as opposed to an in-ground latrine. Access to a project latrine was not associated with additional decreases in diarrhea compared to the “washable toilet”. It is unclear why this group was chosen as the reference group, but its selection precludes understanding how project latrines affected diarrhea compared to no sanitation when adjusting for confounders. Wolf et al. were restrained to report the unadjusted OR with a hand-calculated confidence interval. Due to the differences between intervention and control communities described above, this unadjusted effect estimate has a high risk of bias due to confounding and must be considered with caution.

The other successful latrine intervention was another large-scale national WASH campaign that employed a CLTS-like intervention in rural Mozambique (the One Million Initiative) [90]. The study outcome was self-reported water-related disease for any member of the household, and it was reported with six-month and two-week recall periods. It is not clear how the two recall periods were used in the analysis or which resulted in the estimate reported in the latest review. The control group comprised communities that were located in districts where the intervention was implemented, but control villages themselves were not included in the intervention. Wolf et al. were able to obtain additional information from the authors, but the quantitative effect of the intervention on diarrhea is not shown in the manuscript text and is not readily available in the literature. Thus, we are unable to determine if there are potential limitations to the validity or generalizability of the estimated effect, as we did for the intervention in Honduras. One potential design limitation is the use of pipeline controls, which does not guarantee equal covariates on average, as does randomization. The likely confounding described above in Honduras emphasizes the potential for bias introduced by this method [24].

Both of these trials tested the effectiveness of a national sanitation campaign, whereas the WASH-Benefits trials tested a latrine intervention at the household level. The two arms of this trial found different results, possibly demonstrating that sanitation can prevent diarrhea under the right circumstances. In Bangladesh, a 39% relative reduction in diarrhea might have been achieved in part due to the local population’s receptiveness to behavior change, which possibly lead to higher compliance than in Kenya. That the other effective latrine intervention with trustworthy results was a large national campaign supports the need for intensive efforts to successfully achieve community buy-in and behavior change. Another potential explanation for the discordant results of WASH-Benefits is the lower diarrheal prevalence found in Bangladesh during the study period, which was around 5% in the control group compared to 27% in Kenya [1,4]. Household-level sanitation interventions likely do not effectively prevent transmission from the outside environment. If a setting with lower diarrheal prevalence is also characterized by lower environmental transmission, household-level sanitation may have better success in further reducing diarrhea. A final explanation for these mixed results is that unmeasurable contextual differences between settings, interventions, implementations, and studies critically influence effectiveness. The importance of context further underscores the difficulties in describing the multifaceted research body on sanitation with a single average effect.

The majority of studies ever conducted on sanitation interventions found that latrine interventions do not prevent diarrhea. In light of our review of historical average estimates, this result is not as surprising as it first appears. Instead, the more unique result is that WASH-Benefits Bangladesh, using a latrine intervention, without attempting to achieve high coverage, and employing a study-initiated access model, led to a successful reduction in the prevalence of diarrhea.

### 4.3. Intervention Coverage

Wolf et al. (2018) previously showed that interventions that reach 75% coverage or more in the intended population have a stronger effect on diarrhea than studies reaching lower coverage. We found that excluding sewerage interventions, which inherently reach very high coverage and also have the strongest effects on diarrhea, diminishes this effect. After excluding sewerage interventions, we found no difference between studies above or below 60% coverage. Non-sewerage interventions that achieved very high coverage (above 90%) did have a marginally significant effect on diarrhea (we were unable to use a separate 75% threshold for non-sewerage studies because no study reached coverage between 75% and 90%). But only two studies reached 90% coverage, including a complete water, sanitation, and hygiene intervention conducted in Bangladesh [49] and the likely confounded analysis of a social investment campaign in Honduras [24]. These two studies do not provide strong evidence for the effect of reaching high coverage with a latrine intervention on diarrhea. Observational research has found that community sanitation coverage is related to child height and stunting in Mali and Ecuador [9,10]. One observational study from national survey data in India found that community coverage was related to diarrhea [115], but another observational study found no effect of community coverage on diarrhea in Mali [9]. Additional theoretical model analysis has suggested that all benefits from sanitation interventions come from the indirect effects due to community coverage [116]. There is not enough evidence to know if these latrine interventions could have had a stronger effect at higher coverage, but it is possible that not approaching herd protection was a factor in the observed results of the WASH-Benefits Kenya and SHINE trials.

### 4.4. Study-Initiated vs. Household-Initiated Access

WASH-Benefits Kenya, WASH-Benefits Bangladesh, SHINE, and another trial in India [55] employed a sanitation intervention that was study-initiated, meaning that households were asked to participate in the study with the knowledge that a latrine would be constructed if they agreed. Of these, only WASH-Benefits Bangladesh led to a decrease in diarrhea. In contrast, 12 studies on sanitation access that was household-initiated, meaning members of the household made the decision to obtain sanitation without direct study contact, led to a statistically significant 16% overall reduction in diarrhea. These studies include community interventions in which a sanitation-promoting environment was created, as well as analyses of DHS or national survey data in which households had existing access to sanitation. These results suggest that there is an important difference between households that are self-motivated to obtain sanitation access and households that obtain sanitation access only because it was offered directly.

Survey analyses measure the effect of existing sanitation access on diarrhea, which could be confounded by other household characteristics, such as wealth or education. However, the studies included here all employed some causal-based analysis, such as propensity score matching. These methods reduced the likelihood of bias from analyzing observational data, but it is possible that residual confounding remained. Fundamental differences between self-motivated sanitation access and access provided in randomized trials also could explain why observational studies on sanitation often show an association with diarrhea, while we have found that most RCTs of sanitation interventions do not impact diarrhea. Some of these differences could be due to residual confounding, as pointed out in an observational re-analysis of the WASH-Benefits control groups that found latrine access was associated with improved child growth but credited the association to confounding [117]. Another potential explanation is effect modification due to different levels of motivation to obtain sanitation access. This effect modification could explain why our analysis found a stronger effect for survey analyses and household-initiated access compared to RCTs and study-initiated access, and could be related to the difficulties of achieving behavior change in intervention trials. This modification also could explain the association between baseline latrine access and child growth in the WASH-Benefits re-analysis. Rather than discounting the results of many observational studies, further work should be done to understand the different motivational drivers identified by these studies.

## 5. Conclusions

The results of this review support the message that new forms of transformative WASH must be developed in order to improve health [6]. We found that sanitation interventions have rarely been shown to prevent diarrhea, but this fact was obscured by numerous average estimates that were not limited to studies on sanitation alone and that failed to adequately consider which forms of sanitation were driving results. Given the complexity of any environmental intervention, context matters in its success or failure, and average effects across studies mask those crucial contextual differences. We showed the implications of this for diarrhea. These results likely apply to other health outcomes, including child growth and sub-clinical infection, but an understanding of outcome-specific nuances warrants more attention. We also did not assess the importance of sanitation access for social outcomes potentially related to sanitation, such as dignity, safety, and educational attainment. These factors alone may justify the implementation of basic sanitation improvements in some settings.

This review uncovers important limitations in the existing literature on sanitation and diarrhea, along with opportunities to improve interventions. Transformative sanitation, and WASH more broadly, is not yet defined; but the important study features identified here, including complete separation of waste from the home, high community coverage, and sufficient household motivation, are likely prerequisite characteristics of future transformative sanitation interventions. More work is needed to understand how each of the factors we described is specifically related to transmission and disease. Future research on transformative sanitation must depend on rigorously conducted trials, as well as thorough and carefully controlled observational studies on prevalent sanitation behaviors. Some of this work will require rigorous inquiry from social science disciplines to better understand the interplay between social and environmental contexts. With strengthened foundational research, new forms of transformative sanitation interventions can be developed to prevent diarrhea and achieve better health worldwide.

## Figures and Tables

**Table 1:** History of Literature Reviews on Sanitation and Diarrhea.

Review	Scope of Review	Eligibility Criteria	Number of Studies on Sanitation and Diarrhea	Number of Studies Included in Overall Estimate	Overall Estimate of the Effect of Sanitation on Diarrhea	Conclusions	Limitations
Blum and Feachem, 1983 [13]	Studies on water supply and/or excreta disposal facilities and any health outcome	None	14 studies on excreta disposal (alone or with water supply) and diarrhea	N/A	N/A	Severe methodological limitations in almost all studies raises doubts to the validity of their conclusions	Water supply and excreta disposal were not assessed separately; a health recall period greater than 48 h was considered a methodology problem
Esrey and Habicht, 1986 [14]	Effect of water and sanitation interventions on diarrhea, infection, nutritional status, and childhood mortality	Any study that compared groups with different water and/or sanitation conditions	8 studies on sanitation and water together; 23 other studies on sanitation and some health outcome; 3 studies confirmed to measure sanitation and diarrhea morbidity	N/A	N/A	Sanitation interventions can improve child health, especially when tailored to local communities	Did not clearly distinguish between studies on different health outcomes
Esrey et al. 1991 [11]	Effect of drinking water and sanitation interventions on diarrhea, nutritional status, mortality, and various infections	Studies published after the previous review (1986)	30 studies on sanitation alone; 18 “rigorous” studies did not have severe flaws	11 for all studies; 5 for “rigorous” studies (studies not identified in text)	Median effect of all 11 studies: 22% reduction Median effect of 5 “rigorous” studies: 36% reduction	Despite the poor quality of existing studies, it can be inferred that sanitation improvements lead to better health	The authors do not indicate which studies were “rigorous”, and it is not clear from reviewing the references separately Using the median value hides the potentially wide range of effects, especially for only 5 “rigorous” studies
Fewtrell et al. 2005 [15]	First systematic review of water, sanitation, and hygiene interventions on diarrhea	Studies that measured the effect of a water, sanitation, hygiene, or combined intervention	4 eligible studies	2 [16,17]	32% reduction (RR = 0.68, 95% CI 0.53, 0.87)	Sanitation interventions are effective at reducing diarrhea, although the evidence is limited Few differences between these results and those from Esrey et al. 1991	The two studies used to calculate an overall effect were (i) a sanitation and water supply intervention and their effects on cholera and (ii) a hospital-based case-control study; the two studies not used for the estimate are not identified in the study
Waddington et al. 2009 [18]	Update to Fewtrell et al. 2005	RCTs or studies employing quasi-experimental designs, including matched analysis of survey data	6 studies; 3 high-quality studies	6 studies [19,20,21,22,23,24]	37% reduction (Effect Size (ES) = 0.63, 95% CI 0.43, 0.93)	Sanitation interventions are highly effective at reducing diarrhea, but few studies have been conducted on the topic	The overall “effect estimate” did not attempt to convert effects from different studies to the same ratio (e.g., RR or OR) The estimate included 3 studies of “poor quality”; the three high quality studies included an analysis of DHS data and two studies on sewerage
Clasen et al. 2010 [25]	Systematic review of sanitation interventions on diarrhea using the Cochrane methodology	Randomized, quasi-randomized, or non-randomized controlled trials	13 studies; 7 in Chinese, 5 in English, 1 in French	N/A	N/A	The heterogeneity in type and quality of sanitation interventions is high and does not allow for estimation of an overall effect; but there is evidence that sanitation interventions prevent diarrhea	Confidence intervals were not extracted or reported from 11 studies due to insufficient number of clusters (e.g., a one-to-one village comparison); only point estimates were reported for those studies
Norman et al. 2010 [26]	Systematic review on the effects of sewerage access on diarrhea and enteric infection	Any trial, cohort, case-control, or cross-sectional study	25 total studies; 17 on diarrhea	17 studies on diarrhea [20,21,22,24,27,28,29,30,31,32,33,34,35,36,37,38,39]	30% reduction (RR = 0.70, 95% CI 0.58, 0.85)	Sewerage is associated with reduced diarrhea in all age groups; confounding from observational studies is a potential issue, but sensitivity analyses suggest it is not a major limitation	Depends on observational studies, but the authors attempted to accounted for potential confounding through sensitivity analyses
Cairncross et al. 2010 [40]	The impact of improved water quality, water quantity, and sanitation on diarrhea	First, intervention studies on sanitation and diarrhea After only four studies in Chinese were found, the criteria expanded to include before and after studies	4 quasi-randomized studies published in Chinese and 1 before and after sewerage study	N/A	No overall effect was calculated	The authors noted the consistency of diarrhea reductions found in various reviews of 36% (Esrey et al. 1991), 32% (Fewtrell et al. 2005), 20-51% (the median values of the four Chinese studies), and 22–43% (the one sewerage study, Barreto et al. 2007), although there is a serious lack of evidence on the subject There is not enough evidence to support moving past the consensus estimate of 36% (Esrey et al. 1991)	In finding no studies that fit their original criteria, the authors showed the striking lack of evidence on sanitation and diarrhea The comparison between different effect estimates did not note that one estimate was a single sewerage study, another came from only two studies (Fewtrell et al. 2005), and results from the four Chinese studies ranged from 8 to 63%
Heijnen et al. 2014 [41]	Comparison of shared sanitation vs. household latrine access on diarrhea, infection, enteric fevers, adverse birth outcomes, trachoma, and other fecal-oral diseases	Any study that compared health outcomes of populations using shared sanitation to those using household latrines	9 studies with diarrhea as an outcome measure	12 estimates from 6 studies [27,42,43,44,45,46]	44% increased odds of diarrhea when sharing sanitation (OR = 1.44, 95% CI 1.18, 1.76)	Those relying on shared sanitation are at higher risk of diarrhea and other health outcomes, although the conclusions are limited by methodological concerns, not knowing actual latrine use, and study heterogeneity	The authors acknowledged several limitations of their results, including that none of the studies followed an experimental design and not all studies adjusted for confounding. All studies were hospital- or clinic-based case-control studies
Wolf et al. 2014 [47]	Impact of drinking water and sanitation interventions on diarrhea	RCTs, quasi-randomized and non-randomized control trials, observational studies when based on an intervention, time-series studies, and survey data with causal matching methods	11 total studies; 2 sewerage studies	11 for total effect; 9 for non-sewerage effect [17,19,21,22,24,48,49,50,51,52,53]	All studies: 28% reduction (RR = 0.72, 95% CI 0.59, 0.88) Non-Sewerage Studies: 16% reduction (RR = 0.84, 95% CI 0.77 0.91)	Sanitation interventions can lead to reductions in diarrhea Sewerage interventions might be even more effective, but there were only two studies to reach a conclusion on	Mostly limited by underlying evidence Sewerage was the only factor assessed as a potential effect modifier
Jung et al. 2017 [54]	Comparison of neighborhood and household sanitation access on diarrheal morbidity	Studies that estimated the association between sanitation at the household and/or neighborhood level and diarrhea; excluded studies that aggregated the effect of both levels	22 total studies; 5 neighborhood level; 16 household level; 1 study measured both levels	6 for the effect of neighborhood level; 17 for household level [17,21,38,39,49,55,56,57,58,59,60,61,62,63,64,65,66,67,68,69,70,71]	Neighborhood Sanitation: 44% reduction (OR = 0.56, 95% CI 0.40, 0.79) Household Sanitation: 36% reduction (OR = 0.64, 95% CI 0.55, 0.75)	Both neighborhood level and household level sanitation are independently, and nearly equally, associated with reduced risk of diarrhea	This article reviewed mostly observational research, making it harder to compare to other reviews Neighborhood sanitation effect was partially driven by one sewerage study [21]; the other neighborhood exposures relied on visual inspection for fecal matter or wastewater and were not strong indicators of sanitation
Freeman et al. 2017 [72]	The effect of sanitation interventions on diarrhea, various infections, and nutritional status	Excluded cross-sectional studies with no matching methods	33 studies	27 total studies; 16 intervention studies [17,19,21,22,24,48,49,50,51,52,55,73,74,75,76,77,78,79,80,81,82,83,84,85,86] (could not find a citation for a study listed as Castro 2015)	All studies: 12% reduction (OR = 0.88, 95% CI 0.83, 0.92) Intervention studies: 23% reduction (OR = 0.77, 95% CI 0.66, 0.91)	The studies reviewed were of low quality, but the results indicate an association between sanitation and diarrhea	Studies that went into the total estimate used a wider variety of study designs, including three hospital-based case-control studies Other studies in the overall estimates were unique, including five effect estimates from school-based sanitation interventions
Wolf et al. 2018 [8]	Update to Wolf et al. 2014	RCTs, quasi-randomized and non-randomized control trials, observational studies when based on an intervention, time-series studies, and survey data with causal matching methods	19 studies	22 effect estimates from 19 total studies; 15 non-sewerage studies; 4 sewerage studies [19,21,22,24,48,49,50,51,52,53,55,58,77,78,79,87,88,89,90]	All studies: 25% reduction (RR = 0.75, 95% CI 0.6, 0.88) Non-sewerage studies: 16% reduction (RR = 0.84, 95% CI 0.73, 0.98) Studies with > = 75% coverage: 45% reduction (RR = 0.55, 95% CI 0.34, 0.91) Studies with < 75% coverage: 24% reduction (RR = 0.76, 95% CI 0.51, 1.13)	Evidence is limited, but sanitation is associated with reduced diarrhea, especially with high coverage	Only one coverage threshold was assessed The authors did not note that three out of five studies that achieved coverage over 75% are sewerage studies and may not reflect latrine coverage Studies testing an intervention that included more than sanitation alone were not separated from the overall estimate

**Table 2:** Studies on Sanitation Interventions Included in Sub-Group Meta-Regression Analyses.

	Type of Intervention	Community Coverage	Community-Led Total Sanitation Model	Initiation of Sanitation Access	Effect on Diarrhea (95% CI)	Notes
Aziz et al. 1990 [49]	Interventions of More Than Sanitation Alone	92%	No	NA or Unknown	0.74 (0.69, 0.80)	A community-based water, sanitation, and hygiene intervention was associated with a 26% reduction in diarrheal disease in children in rural Bangladesh.
Begum et al. 2011 [50]	None: Analysis of National Survey or DHS Data	Not Reported	No	Household	0.85 (0.63, 1.13)	An analysis of DHS and MICS survey data from Bangladesh found that sanitation had no association with diarrheal disease in children, unless the household had both improved sanitation and improved water access.
Bose 2009 [19]	None: Analysis of National Survey or DHS Data	Not Reported	No	Household	0.64 (0.45, 0.89)	A propensity score matched analysis of DHS data from 2006 in Nepal found that access to improved sanitation reduced childhood diarrhea by 46%.
Briceño et al. 2015 [87]	Latrine Intervention	56%	Yes	Household	0.99 (0.75, 1.30)	An RCT of a large-scale, government-led, community-based handwashing and sanitation campaign found no effect on diarrhea in rural Tanzania. There was a statistically significant reduction in diarrhea only among communities that received both interventions, and only at the 10% confidence level.
Capuno et al. 2012 [51]	None: Analysis of National Survey or DHS Data	Not Reported	No	Household	1993: 0.85 (0.62, 1.15) 1998: 0.89 (0.65, 1.21) 2003: 0.80 (0.60, 1.06) 2008: 0.69 (0.45, 1.01)	A propensity score analysis of four years of DHS data in the Philippines reported a 10 percentage point decrease in diarrheal incidence associated with access to a flush toilet. But this value is the maximum difference in one of the four years (2008) from six different matching methods. It is not clear which matching method was recorded for Wolf et al. (2018).
Clasen et al. 2014 [55]	Latrine Intervention	38%	No	Study	0.97 (0.84, 1.13)	An RCT of a community-based sanitation promotion and construction intervention found no association with diarrheal disease in Odisha (Orissa), India.
Fan and Mahal 2011 [52]	None: Analysis of National Survey or DHS Data	Not Reported	No	Household	1.07 (0.88, 1.29)	Several matched analyses were conducted using 1994 survey data from India. Improved toilets were associated with an 8.5 percentage point reduction in diarrhea using exact matching, but no association was found using two other matching methods.
Garrett et al. 2008 [58]	Interventions of More Than Sanitation Alone	49%	No	Household	0.31 (0.23, 0.41)	A village-level RCT on a combined water access, water treatment, latrine promotion, and behavior change intervention found that living in an intervention village was associated with a 69% reduction in diarrhea. This is the value reported by Wolf et. al., but includes all of the interventions together. Latrine presence was independently associated with diarrhea (RR = 0.71, 95% CI 0.54, 0.92).
Godfrey et al. 2014 [90]	Latrine Intervention	62%	Yes	Household	0.54 (0.29, 1.01)	An RCT was implemented to test the effect of a large-scale government WASH program in Mozambique (The One Million Initiative). A water intervention, a CLTS intervention, and a water + CLTS intervention group were compared to controls. Controls were from districts where the government had begun implementing the intervention, but it was not implemented in the control communities themselves. The intervention was implemented in communities and in schools. The outcome, “self-reported water-related disease”, was measured for all age groups. This outcome was measured with 6-month and 2-week recall in a household questionnaire. Water-related disease decreased in all groups, including the control group, and decreased the most in the CLTS-only group. Outcome rates are not presented in the available text; rates on only presented graphically. Wolf et al. received additional information from the author.
Khush and London 2009 [88]	Interventions of More Than Sanitation Alone	57%	No	Household	1.00 (0.43, 2.32)	A non-randomized CLTS and drinking water improvement campaign in India did not result in changes to diarrheal disease, but the prevalence of diarrhea in all groups was low (2%).
Klasen et al. 2012 [77]	Sewerage Intervention	85%	No	NA or Unknown	0.81 (0.35, 1.90)	The effect of extending access to piped water and sewerage in urban Yemen was estimated in two regions: a costal region and a mountain region. Diarrheal risk increased in the mountain region after the intervention, while risk decreased in the coastal region. The intervention is a drinking water and sewerage intervention, compared to a control group that only received the drinking water intervention.
Kumar and Vollmer 2012 [53]	None: Analysis of National Survey or DHS Data	Not Reported	No	Household	0.82 (0.79 0.85)	A propensity score analysis of survey data in India found no effect of improved sanitation among low- and middle-income households or for girls; there were effects for high income households and boys. The statistically significant effects are each 2–3 percentage point reductions.
Messou et al. 1997 [48]	Interventions of More Than Sanitation Alone	Not Reported	No	NA or Unknown	0.71 (0.56, 0.92)	Study was published in French. The intervention was a shared (public) double pit latrine, designed to be shared by 10 people, along with improved water supply, hygiene promotion, and oral hydration therapy (this information was extracted from Clasen et al. 2010)
Moraes et al. 2003 [21]	Sewerage Intervention	91%	No	NA or Unknown	0.31 (0.28, 0.34)	Neighborhoods that received government expanded sewerage access had almost 70% fewer episodes of diarrhea compared to control neighborhoods. Analysis was adjusted for child′s age, gender and birth order, number of children aged < 5 years in the household, crowding, mother′s education, monthly per capita income, exclusive use of kitchen, animals in the house, presence of a washstand, water usage and house floor material.
Patil et al. 2014 [78]	Latrine Intervention	41%	Yes	Household	0.97 (0.78, 1.22)	An RCT of a community-based sanitation intervention (TSC) in rural India found no health benefits, including diarrheal disease.
Pickering et al. 2015 [79]	Latrine Intervention	65%	Yes	Household	0.93 (0.76, 1.14)	An RCT of a community-based sanitation intervention (CLTS) in rural Mali found no differences between intervention and control villages on diarrheal disease. Intervention children were taller and less likely to be stunted.
Pradhan and Rawlings 2002 [22]	Sewerage Intervention	100%	No	NA or Unknown	0.43 (0.11, 1.71)	An analysis of a multi-faceted social investment project in Nicaragua found no association between sewerage promotion and diarrhea in children under six. Not all households in the intervention area were connected to the sewer network. There were only 23 children under six in the intervention group; two of the 23 were reported to have diarrhea. The effect estimate differs from that recorded in a review of sewerage studies, (Norman et al. 2010), where RR = 0.37 (95% CI 0.20, 0.66). It is not clear from either review or the article text why these numbers differ or which is a more accurate representation of the effect.
Roushdy et al. 2012 [89]	None: Analysis of National Survey or DHS Data	63%	No	Household	1.42 (0.76, 2.68)	An analysis of DHS data from 2008 in Egypt found that improved sanitation had a positive, non-significant association with diarrheal disease in children.
Walker et al. 1999 [24]	Latrine Intervention	90%	No	NA or Unknown	0.65 (0.47, 0.90)	This study evaluated a mostly World Bank/Honduran government funded social investment project in Honduras in the 1990s. Municipalities were offered projects from a “menu” of options. It is not clear if municipalities chose only one project or any projects that could be afforded by their allotted budget. The estimate reported by Wolf et al. is a crude estimate comparing intervention households to those who would soon receive the intervention (pipeline controls). In their executive summary, Walker et al. state that confounding is a large concern since pipeline controls were more rural, had worse sanitation, were less educated, and had lower incomes compared to intervention households. It is also not clear if the control group comprised municipalities that had chosen latrine projects or those that had not chosen their project(s). Full article text only found in Spanish; an executive summary is available in English.
Humphrey et al. 2019 [2]	Interventions of More Than Sanitation Alone	Not Reported	No	Study	1.18 (0.87, 1.61)	The Sanitation Hygiene Infant Nutrition Efficacy (SHINE) trial was a randomized controlled trial of a combined water, sanitation (construction of a ventilated improved pit latrine), and hygiene intervention. The intervention had no effect on diarrhea in children.
Luby et al. 2018 [4]	Latrine Intervention	Not Reported	No	Study	0.61 (0.46, 0.81)	The WASH-Benefits-Bangladesh trial was a randomized controlled trial that included a sanitation arm (compound level pour flush latrine construction). The sanitation intervention led to a reduction in diarrhea in children, from 5.7% to 3.5% using one-week recall.
Null et al. 2018 [1]	Latrine Intervention	Not Reported	No	Study	0.99 (0.88, 1.10)	The WASH-Benefits-Kenya trial was a randomized controlled trial that included a sanitation arm (compound level improved latrines). The intervention had no effect on diarrhea in children.

**Table 3:** Results of Subgroup Meta-Regression Models.

Model	Risk Ratio (95% CI)	Number of Studies Included (Number of Estimates)
All Studies	0.80 (0.67, 0.92)	22 (25)
*Intervention Type*		
Latrine interventions	0.90 (0.67, 1.12)	8 (8)
Interventions on more than sanitation alone	0.74 (0.46, 1.02)	5 (5)
Sewerage interventions	0.36 (0.00, 0.76)	3 (3)
No Intervention: National survey or DHS analysis	0.85 (0.66, 1.04)	6 (9)
*Other Sub-Groups*		
Community-led total sanitation studies	0.91 (0.55, 1.28)	4 (4)
Household-initiated WASH access ^1^	0.84 (0.68, 1.00)	12 (15)
Study-initiated interventions ^2^	0.95 (0.67, 1.24)	4 (4)

^1^ Includes studies in which the household chose to obtain access without direct contact from a study team, including some sanitation promotion interventions and cross-sectional surveys. ^2^ Includes studies in which households were asked to participate knowing that a latrine would be constructed if they agreed.

**Table 4:** Effect Modification by Sanitation Coverage.

Model	Risk Ratio (95% CI)	Number of Studies Included
*All Studies*		
Under 60% Coverage	0.85 (0.54, 1.17)	5
Over 60% Coverage	0.65 (0.42, 0.88)	8
Under 75% Coverage	0.88 (0.61, 1.15)	8
Over 75% Coverage	0.56 (0.30, 0.82)	5
Under 90% Coverage	0.88 (0.62, 1.14)	9
Over 90% Coverage	0.55 (0.28, 0.82)	4
*Excluding Sewerage Intervention Studies*		
Under 60% Coverage	0.85 (0.54, 1.17)	5
Over 60% Coverage	0.80 (0.51, 1.08)	5
Under 75% Coverage ^1^	0.88 (0.61, 1.15)	8
Over 90% Coverage ^1^	0.72 (0.37, 1.07)	2

^1^ The two non-sewerage studies that reached 75% coverage also reached over 90% coverage, so the 75% threshold could not be assessed separately for these studies.
